# Association of C1q/TNF-Related Protein-3 (CTRP3) and CTRP13 Serum Levels with Coronary Artery Disease in Subjects with and without Type 2 Diabetes Mellitus

**DOI:** 10.1371/journal.pone.0168773

**Published:** 2016-12-29

**Authors:** Reza Fadaei, Nariman Moradi, Mehdi Baratchian, Hassan Aghajani, Mojtaba Malek, Ali Akbar Fazaeli, Soudabeh Fallah

**Affiliations:** 1 Department of Biochemistry, Faculty of Medicine, Iran University of Medical Sciences, Tehran, Iran; 2 Department of Clinical Biochemistry, Faculty of Medicine, Tehran University of Medical Sciences, Tehran, Iran; 3 Department of Cancer Biology, Lerner Research Institute, Cleveland Clinic, Cleveland, Ohio, United States of America; 4 Interventional Cardiology Department, Tehran Heart Center, Tehran University of Medical Sciences, Tehran, Iran; 5 Research Center for Prevention of Cardiovascular Disease, Institute of Endocrinology & Metabolism, Iran University of Medical Sciences, Tehran, Iran; 6 Research Center of Pediatric Infectious Disease, Rasool Akram Hospital, Iran University of Medical Sciences, Tehran, Iran; East Tennessee State University, UNITED STATES

## Abstract

C1q/TNF-Related Protein-3 (CTRP3) and CTRP13 are two newly discovered adipokines regulating glucose and lipid metabolism. But their role in type 2 diabetes mellitus (T2DM) and coronary artery disease (CAD) is still in infancy. The aim of this study was to investigate the associations of gene expression and serum levels of CTRP3 and CTRP13 with CAD, metabolic and inflammatory markers in patients with and without T2DM. Serum levels of CTRP3, CTRP13, adiponectin and inflammatory cytokines and their gene expression in peripheral blood mononuclear cells (PBMCs) were determined in 172 subjects categorized as group I (without T2DM and CAD), group II (with CAD but no T2DM), group III (with T2DM but no CAD) and group IV (with T2DM and CAD). Serum levels and gene expression of CTRP3, CTRP13 and adiponectin in the group I were higher compared to other groups. Inflammatory cytokines in the control group were lower than other groups too. CTRP3 serum levels have an independent association with BMI, smoking and CTRP3 gene expression; also CTRP13 serum levels has an independent association with BMI, HDL-C, insulin, HOMA-IR, HbA1c and TNF-α. Decreased serum levels of CTRP3 and CTRP13 were also associated with CAD. It appears that the decreased levels of CTRP3 and especially CTRP13 were associated with increased risk of T2DM and CAD. These findings suggest an emerging role of these adipokines in the pathogenesis of CAD, but further studies are necessary to establish this concept.

## Introduction

Adipose tissue is recognized as the largest endocrine organ in the body that secretes various adipokines such as tumor necrosis factor-alpha (TNF-α), resistin, visfatin and leptin [1]. Adipokines are involved in regulating glucose metabolism, insulin signaling pathway, lipid and lipoproteins metabolism and inflammation, which in this way interact with the pathogenesis of type 2 diabetes mellitus (T2DM), metabolic syndrome and atherosclerotic cardiovascular disease [2]. Circulating levels of adipokines are mostly dysregulated in the metabolic disorders and obesity [1]. Among adipokines secreted from adipose tissue, adiponectin is one of the most potent molecules with respect to anti-atherosclerotic, anti-inflammatory and insulin-sensitizing activities [2,3], however adiponectin-deficient animal models display modest phenotype [4]. This discrepancy suggests that a compensatory effect may be caused by the family of C1q TNF-related proteins (CTRP) [4,5]. The CTRP family is a newly discovered and highly conserved paralogue of adiponectin, and comprises 15 members (CTRP1-CTRP15) [6–8]. Despite structural similarities between CTRP family and adiponectin, they exert pleiotropic effects on cell metabolism and have different regulation patterns [6].

CTRP3 (also known as CORS-26, cartducin and cartonectin) is a member of this family [5]. There is evidence that CTRP3 level has a negative association with leptin levels [9]. CTRP3 also reduced gluconeogenesis and subsequent glucose output in hepatocytes [9]. Also, this adipokine has cardio-protective properties [10] and its circulating levels drop in obesity and individuals with high blood pressure, and is inversely associated with insulin resistance parameters [11]. This protein was found to inhibit inflammation and improve insulin sensitivity in 3T3-L1 adipocytes [12]. Studies on serum levels of CTRP3 in patients with diabetes are contradictory. A study by Choi et al., reported increase of CTRP3 levels in diabetes [13], but another study showed decrease in CTRP3 levels [14]. Also, there is a lot of conflicting data regarding the associations between CTRP3 levels and obesity [15,16]. CTRP13 is another member of the CTRP family, which is mainly expressed in adipose tissue and can increase insulin-mediated glucose uptake and reduce gluconeogenesis [17]. This protein has also a crucial role in regulating food intake and body weight [18]. But so far it has not been studied in the conditions of T2DM and CAD. Although there is limited number of data supporting alteration in CTRP3 metabolic disorders, no study has specifically assessed the association of CTRP13 circulating levels and peripheral blood mononuclear cells (PBMCs) gene expression with metabolic status in T2DM patients.

Therefore, in this study we investigated the serum levels and PBMCs gene expression of CTRP3 and CTRP13 in patients with and without T2DM and their association with related metabolic and inflammatory markers.

## Subjects and Methods

### Study populations

This case-control study was conducted on 172 subjects aged between 45–75 years, who underwent coronary angiography at Rasoul-e-Akram Hospital (Tehran, Iran). Written consent was taken from all subjects. The study was in accordance with the Declaration of Helsinki and approved by the Ethics Committee of the Iran University of Medical Sciences. The subjects were divided into 4 groups equally (each group 43 persons). Group I (Control): subjects without T2DM and without CAD, group II (CAD): patients with CAD and without T2DM, group III (T2DM): patients with T2DM and without CAD and group IV (CAD+T2DM): patients with T2DM and CAD. CAD was diagnosed by cardiologist based on coronary angiography results. Subjects who had coronary artery luminal reduction ≥50% in at least one coronary vessel were diagnosed as CAD (group II and group IV). CAD severity was defined using the number of vessels that showed ≥50% reduction in angiography imaging. Subjects with <30% stenosis of coronary artery in angiography were considered as Non-CAD and were included in group I and group III. Also, subjects who had carotid plaque, unstable angina and any history of cardiovascular disease, including acute coronary syndrome, cerebrovascular, coronary artery and peripheral artery disease were excluded from Non-CAD subjects (group I and group III). T2DM was diagnosed based on American Diabetes Association (ADA) criteria [19]. Those with a history and evidence of myocardial infraction, stroke, kidney disease, cancer, autoimmune diseases, chronic inflammation and treated with thiazolidinedione family drugs were excluded from study. Using thiazolidinedione was an exclusion criteria because the CTRP3 promoter have a peroxisome proliferator-activated receptor (PPAR) response element and also upregulate expression of CTRP13 and other CTRP family member [17,20], Subjects received no glucagon-like peptide-1 receptor agonists which upregulate CTRP3 expression [21]. Arterial hypertension was defined as SBP≥140mm Hg, DBP≥90mm Hg in repeat measurement or current use of antihypertensive medication. Individuals with the history of smoking in the last three months were considered as a smoker.

### Anthropometric measurement and laboratory assessment

A standard and self-reported questionnaire was used for each participant to record demographic and medical history, as well as medication treatment (S1 Questionnaire). Body mass index (BMI) was calculated by standard formula (weight (kg)/height (m^2^)). Systolic and diastolic blood pressure were measured by a standard sphygmomanometer and in the sitting position after resting for 5 minutes. Waist circumference (WC) was measured by a soft tape at the midpoint between costal margin and iliac crest, while the subject was at the end of a normal expiration and hip measured at the maximum circumference of the buttocks. Waist-to-hip ratio (WHR) was calculated as waist circumference (cm) divided by hip circumference (cm). Insulin was assessed with ELISA kit (Monobind Inc., USA, Cat# 5825–300). Fasting serum levels of triglycerides (TG), total cholesterol (TC), high-density lipoprotein cholesterol (HDL-C), low-density lipoprotein cholesterol (LDL-C), fasting blood glucose (FBG), creatinine (Cr), alanine amino transferease (ALT) and aspartate amino transferease (AST) were measured using commercially available kits (Pars Azmoon, Iran). For calculating homeostasis model assessment of insulin resistance (HOMA-IR) we used the following equation: [fasting blood glucose (mg/dL)] × [fasting blood insulin (μU/mL)/405].

### Measurement of circulating adipokines and cytokines

Ultrasensitive ELISA kits were used for measuring serum levels of inteleukin-6 (IL-6) (R & D Systems, USA, Cat# HS600B) and TNF-α (R & D Systems, USA, Cat# DTA00C). Also, ELISA kits were used for measuring serum levels of CTRP3 (Adipogen, South Korea, Cat# AG-45A-0042EK-KI01), Adiponectin (Adipogen, South Korea, Cat# AG-45A-0001YEK-KI01) and CTRP13 (AvisceraBioscience, USA, Cat# SK00333-06). Intra-assay coefficients of variation (CV) were 7.3 for CTRP3, 6 for CTRP13, and 3.4 for Adiponectin. Also, Inter-assay CVs were 5.8 for CTRP3, for 10 for CTRP13 and 4.3 for Adiponectin.

### Quantitative PCR

PBMCs isolated from 5ml EDTA blood using ficoll-hypaque (Lympholyte-H; Cedarlane, Canada) gradient centrifugation method as previously described [22]. Total RNA was extracted using a commercial RNA extraction kit (GeneAll Biotechnology, South Korea) and its protocol. 1μg RNA converted to cDNA by a commercial cDNA synthesis kit (Thermo scientific, Fermentas, USA). For gene expression analysis of the target genes, real time PCR was performed using Rotor Gene real-time thermocycler (QIAGEN, Serial number 110530) and SYBR Premix Ex Taq II (Takara Biotechnology, Japan) based on the manufacturer's protocol. β-actin was used as housekeeping gene and data analyzed by the 2^-ΔCt^ method [23]. The sense and anti-sense primer sequences of the targets genes were as follow: β-actin 5´-AAGAGAGGCATCCTCACCCT-3´ and 5´-TACATGGCTGGGGTCTTGAA-3´; CTRP3 5´-GAGTCTCCACAAACCGGAGG-3´ and 5´-TCACCTTTGTCGCCCTTCTC-3´; CTRP13 5´-GAGTTGTGGTGGTGGCAAAG-3´ and 5´-GTTCGCACCAACTTTCCGTC-3´; adiponectin 5´- AGAAAGGAGATCCAGGTCTTATTGGT-3´ and 5´-AACGTAAGTCTCCAATCCCACACT-3´; IL-6 5´- GGTACATCCTCGACGGCATCT-3´ and 5´-GTGCCTCTTTGCTGCTTTCAC-3´; TNF-α 5´- CCCAGGCAGTCAGATCATCTTC-3´ and 5´-AGCTGCCCCTCAGCTTGA-3´.

### Statistical analysis

Categorical data were expressed as frequencies and percentages and compared with χ^2^ test. Quantitative data were tested by Shapiro-Wilk for normality. Normal distributed variables were shown as mean ± standard error of means (SEM) and data with non-normal distribution were shown as median ± interquartile ranges (IQR). Normal distributed variables were compared using Student's *t*-test and one-way ANOVA with the Scheffé’s post hoc analysis and non-normal distributed variables were tested using Mann–Whitney U test and Kruskal-Wallis test and Bonferroni correction was used for post hoc analysis. Then, ANCOVA analysis was performed to remove effect of potential confounders. Pearson correlation analysis was conducted for detecting correlation between continuous variables and spearman correlation used for categorical variables. Before correlation analysis, logarithmic transformation was performed for non-normal distributed variables. To identify independent determinants of the mean CTRP3 and CTRP13 serum levels, first, we conducted univariate linear regression analysis and then the variables with a significant p value were included in the multivariate linear regression models. The variable was considered to be collinear, if variance inflation factor exceeded 10. Logistic regression was conducted with the presence of CAD as dependent variable in three different conditions for non-T2DM subjects (group I and II), T2DM patients (group III and IV), and all participants. Additional logistic regression was conducted in CAD patients (group II and IV) with 3-vessels disease as dependent variables to find out associations between CTRP3 and CTRP13 serum levels with CAD severity.

## Results

### Clinical and laboratory characteristics of the study population

The number of women in group III was higher than other groups. Smokers, subjects with hypertension and antihypertensive medications and statins in groups II, III and IV were higher than the control group. Also no subjects without T2DM (group I and II) took anti-diabetes drugs. The amount of FBG, Insulin, HOMA-IR and HbA1c in T2DM patients (group III and IV) were higher compared to the non-T2DM groups (group I and II). The TG and BMI in the group I were lower than the other groups. Serum levels of TC, LDL-C and Cr in Group IV were higher than the control group. Patients with T2DM (group III and IV) had significantly higher WHR in compared to the control group, however, it was found that subjects in groups II, III and IV had lower HDL-C levels. In detail, HDL-C levels in group IV were also significantly lower compared with group II. However, age, SBP, DBP and serum levels of AST, ALT had no significant difference among four studied groups (Table 1).

**Table 1:** Clinical Biochemical Characteristics of Study population.

Variables		Group I (n = 43)	Group II (n = 43)	Group III (n = 43)	Group IV (n = 43)	p value
		Control	CAD	T2DM	CAD+T2DM	
Sex [male (%)]		26 (60.5)	30 (69.8)	19 (44.2)	32 (74.4)	**0.021**
Age (year)		57.21 ± 1.28	60.91 ± 0.88	59.21 ± 1.18	61.33 ± 1.33	0.060
BMI (kg/m^2^)		24.10 ± 0.47	26.74 ± 0.50^a^^**^	27.32 ± 0.57^b^^**^	27.79 ± 0.61^c^^***^	**<0.001**
WHR		0.91 ± 0.01	0.93 ± 0.01	0.94 ± 0.01^b^^*^	0.95 ± 0.01^c^^*^	**<0.001**
Smoker [n (%)]		8 (18.6)	18 (41.9)	12 (27.9)	22 (51.2)	**0.008**
Hypertension [n (%)]		11 (25.6)	22 (51.2)	16 (37.2)	28 (65.1)	**0.002**
SBP (mm Hg)		130 (98–135)	133 (105–143)	130 (98–142)	133 (108–147)	0.204
DBP (mm Hg)		79 (60–85)	87 (60–95.5)	80 (60–92)	93.47 (76–95.5)	0.120
FBG (mg/dl)		92 (73–95)	94 (74–103.5)	149 (118–172.5) ^b^^***^^,^ ^d^^***^	138 (117–158) ^c^^***^^,^ ^e^^***^	**<0.001**
Triglyceride (mg/dl)		119.02 ± 6.30	155.81 ± 4.41^a^^*^	155.56 ± 7.72^b^^*^	170.56 ± 10.74^d^^***^	**<0.001**
Total Cholesterol (mg/dl)		160.53 ± 5.05	186.05 ± 7.48	182.86 ± 6.43	188.53 ± 7.40^c^^*^	**0.017**
LDL-C (mg/dl)		95.81 ± 4.88	116.07 ± 5.19	107.33 ± 5.36	119.77 ± 5.01^c^^*^	**0.006**
HDL-C (mg/dl)		46.5 ± 0.8	41.9 ± 0.7^a^^***^	41.4 ± 0.6^b^^***^	38.9 ± 0.7^c^^***^^,^ ^e^^*^	**<0.001**
Insulin (μU/ml)		2.6 (1–4.3)	3.8 (0.9–5.5)	11.2 (3.4–12.3)^b^^***^^,^^d^^***^	10 (4.2–12.9)^c^^***^	**<0.001**
HbA1c (%)		4.3 ± 0.14	4.7 ± 0.13	8.0 ± 0.16 ^b^^***^^,^^d^^***^	7.8 ± 0.16 ^c^^***^^,^^e^^***^	**<0.001**
HOMA-IR		0.6 (0.2–1.01)	0.86 (0.16–1.38)	3.86 (1.27–5.41)^b^^***^^,^^d^^***^	3.51 (1.23–5.31)^c^^***^^,^^e^^***^	**<0.001**
Creatinine (mg/dl)		1.17 ± 0.03	1.22 ± 0.03	1.23 ± 0.03	1.31 ± 0.03 ^c^^*^	**0.022**
AST (U/l)		17 (13–20.5)	19 (15–27)	20 (17–25)	20(14–28)	0.095
ALT(U/l)		15 (10–20)	16 (11–27)	18 (12–24)	18 (13–27)	0.147
IL-6 (pg/ml)		5.01 ± 0.21	6.10 ± 0.29	6.53 ± 0.33^b^^**^	8.06 ± 0.36 ^c^^***^^,^ ^e^^***^^,^^f^^**^	**<0.001**
TNF-α (pg/ml)		24.1 (14.3–27.9)	25.4 (24–27)	27.4 (19.6–32.4)	28.2 (23.4–36.5)^c^^**^^,^^e^^**^	**0.004**
Adiponectin (μg/ml)		12.31 ± 0.56	9.86 ± 0.44^a^^**^	10.14 ± 0.42^b^^*^	9.39 ± 0.37^c^^***^	**<0.001**
CTRP3 (ng/ml)		305.3 ± 13.1	240.5 ± 7.3^a^^*^	268.2 ± 10.3	255.4 ± 9.9 ^c^^*^	**<0.001**
CTRP13 (ng/ml)		279 ± 7.7	215 ± 4.9 ^a^^***^	243 ± 4.3 ^b^^***^^,^^d^^**^	192 ± 5.1^c^^***^^,^^e^^***^^,^^f^^***^	**<0.001**
Antihypertensive medication [n (%)] §		5 (11.6)	17 (39.5)	6 (14.0)	22 (51.2)	**<0.001**
Statin use [n (%)] #		8 (18.6)	17 (39.5)	14 (32.6)	23 (53.5)	**0.008**
Oral hypoglycemic agent [n (%)] ¶		-	-	22 (51.2)	28 (65.1)	**<0.001**
Insulin ± Oral hypoglycemic agent [n (%)]		-	-	13 (30.2)	11 (25.6)	**<0.001**
Angiography	1-Vessel		15		12	
	2-Vessel		13		14	
	3-Vessel		15		17	

a: Comparison between Control and CAD

b: Comparison between Control and T2DM

c: Comparison between Control and T2DM+CAD

d: Comparison between CAD and T2DM

e: Comparison between CAD and T2DM+CAD and

f: Comparison between T2DM and CAD+T2DM.

* P < 0.05

** P < 0.01

*** P < 0.001.

§ Antihypertensive medication: mainly angiotensin-converting enzyme inhibitors (captopril, enalapril or lisinopril), with the addition of calcium antagonists or angiotensin II receptor antagonists (losartan) in some patients.

# Statins: mainly simvastatin and pravastatin.

¶ Oral hypoglycemic agent: mainly metformin and sulphonylureas.

### PBMCs gene expression and serum levels of cytokines and adipokines

**Fig 1:**
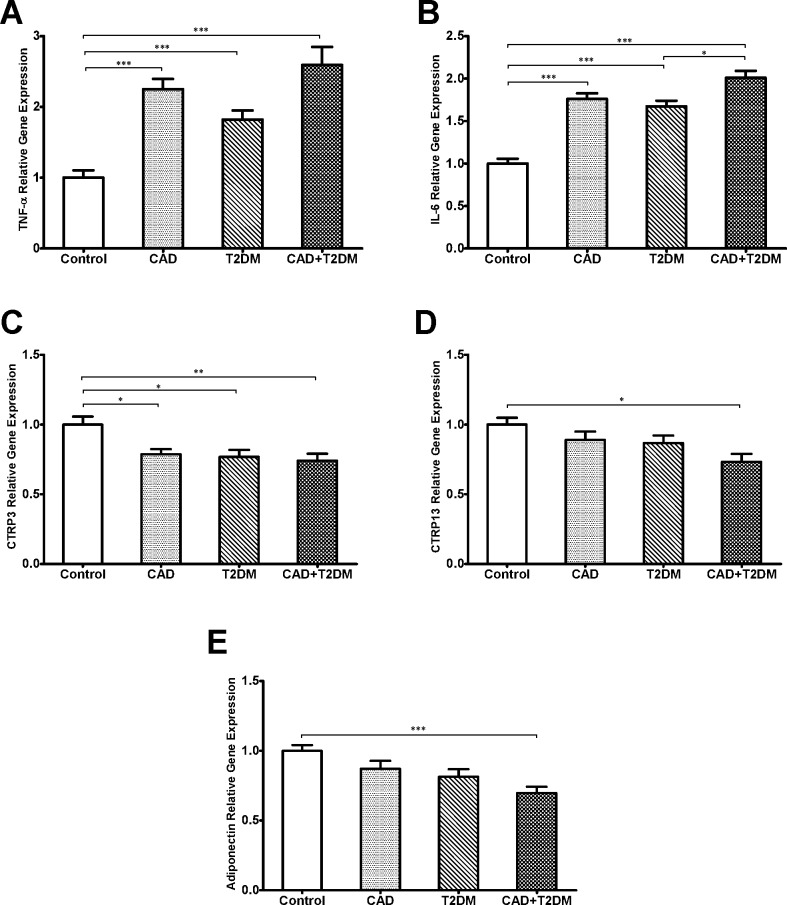
PBMCs gene expression of TNF-α, IL-6, CTRP3, CTRP13 and adiponectin. (A) PBMCs gene expression of TNF-α was higher in groups II, III and IV compared to the group I (all, p < .001). (B) IL-6 PBMCs gene expression was higher in groups II, III, IV compared with the group I (all, p < .001), Also gene expression of IL-6 was higher in group IV compared with group III (p < .05). (C) PBMCs gene expression of CTRP3 was higher in control group compared with CAD group (p < .05), T2DM (p < .05) and CAD+T2DM (< .01). (D) PBMCs gene expression of CTRP13 were higher in control group compared to the CAD+T2DM group (p < .05). (E) PBMCs gene expression of adiponectin was decreased in group IV compared with group I (p < .001).

Gene expression of TNF-α and IL-6 showed a significant increase in groups II, III and IV compared with the control group (p < .001) (Fig 1A and 1B, respectively). Specifically, the IL-6 gene expression was increased in group IV compared to group II (p < .01) (Fig 1B). We also found that CTRP3 expression in groups II, III and IV was decreased compared to group I (p < .05, p < .05 and p < .01, respectively) (Fig 1C) and after adjusting for sex, age, medication, all differences remained significant. Similarly, CTRP13 and adiponectin gene expressions in the group IV were lower compared with the control group (p<0.05 and p<0.001, respectively) (Fig 1D and 1E, respectively). Significant difference for CTRP13 gene expression remained significant after adjusting for sex, age, BMI, medication. Serum levels of TNF-α in group IV compared to subjects without T2DM (group I and II) were increased significantly (both p < .01). Serum levels of IL-6 in group IV were significantly increased compared with the other groups (p < .001 compared with group I and II, and p < .01 for group IV), also increased serum levels was observed in group III compared with group I (p < .01). CTRP3 serum levels in patients with CAD (group II and IV) were significantly lower than the control group (both p < .05) and after adjusting for sex, age, BMI, medication and adiponectin serum levels, only the difference between control and CAD groups remained significant (p = 0.016). Serum levels of CTRP13 displayed a significant reduction in group IV than the other three groups (for all three groups, p < .001). CTRP13 serum levels in group IV compared with group II and I also was significantly lower (p < .001 and p < .01, respectively), and group II showed a significant reduction compared to the group III (p < .001). Also, Serum levels of adiponectin in groups II, III and IV were significantly lower than the control group (p < .01, p < .05 and p < .001, respectively). After adjusting for the effects of sex, age, BMI, medication and adiponectin serum levels on CTRP13 serum levels all differences remained significant. Difference of gene expression and serum levels of CTRP3 and CTRP13 were compared between male and female subjects and we found no significant difference in gene expression and serum levels of CTRP3 or CTRP13.

### Analysis of the association of CTRP3 and CTRP13 serum levels with biochemical and metabolic parameters

Correlation analysis showed a significant positive correlation of circulating levels of CTRP3 with gene expressions of CTRP3 (r = .21, p = .005), adiponectin (r = .15, p = .048) and CTRP13 (r = .15, p = .042).We also found a negative correlation between serum levels of CTRP3 and BMI (r = -.25, p = .001), and smoking (r = -.16, p = .037 (Table 2). Moreover, CTRP13 serum level had significant positive correlation with CTRP13 gene expression (r = .16, p = .035), HDL-C (r = .30, p < .001), adiponectin serum levels (r = .21, p = .005), CTRP3 serum levels (r = .15, p = .042).However, this adipokine demonstrated a negative correlation with BMI (r = -.37, p < .001), WHR (r = -.19, p = .014) SBP and DBP (both, r = -.21, p = .006), TC (r = -.16, p = .042), LDL-C (r = -.24, p = .001), TG (r = -.23, p = .003), FBG (r = -.29, r < .001), insulin (r = -.28, p < .001), HOMA-IR (r = -.29, p < .001), HbA1c (r = -.31, p < .001), IL-6 serum levels (r = -.24, p = .002), serum levels of TNF-α (both, r = -.34, p < .001) and ALT (r = -.17, p = .024) (Table 3).

**Table 2:** Univariate and multivariate analysis of the association between CTRP3 serum levels with correlated parameters.

Variables	Univariate Analysis		Multivariate Analysis	
	β	P value	β	P value
BMI	-4.78	**0.001**	-3.40	**0.027**
WHR	-2.67	0.024	-93.7	0.453
Smoking	-26.8	**0.018**	-22.9	**0.037**
Adiponectin serum levels	3.42	0.048	1.81	0.306
CTRP3 Gene expression	1855	**0.005**	1449	**0.025**
CTRP13 serum levels	0.22	0.042	0.024	0.835

β:unstandardized coefficient

**Table 3:** Univariate and multivariate analysis of the association between CTRP13 serum levels with correlated parameters.

Variables	Univariate Analysis		Multivariate Analysis	
	β	P value	β	P value
BMI	-4.73	**<0.001**	-2.68	**0.003**
SBP^a^	-186	0.006	69.83	0.510
DBP^a^	-140	0.006	-142.7	0.076
Triglyceride	-0.21	0.003	-0.07	0.248
Total Cholesterol	-0.16	0.042	0.34	0.120
LDL-C	-0.35	0.001	-0.60	0.143
HDL-C	2.79	**<0.001**	1.41	**0.034**
FBG^a^	-123	<0.001	-15.7	0.387
Insulin^a^	-39.1	<0.001	-6.97	0.285
HOMA-IR^a^	-31.2	**<0.001**	-14.9	**0.049**
HbA1c	-7.85	**<0.001**	-3.79	**0.040**
TNF-α^a^	-115	**<0.001**	-84.9	**0.001**
IL-6	-5.20	0.002	0.58	0.716
Adiponectin serum levels	3.33	0.005	0.87	0.400
CTRP3 serum levels	0.155	0.042	0.03	0.523
CTRP13 gene expression	4483	0.013	1711	0.277

β: unstandardized coefficient

a: logarithmic transformation was performed

Using univariate linear regression analysis, we confirmed all above-mentioned correlations, and then we included these variables in multivariate linear regression. In all participants BMI, CTRP3 gene expression and smoking were independent predictors of CTRP3 serum levels (all p < .05) (Table 2). Also, based on this analysis, BMI (p = .003), HOMA-IR (p = .049), HbA1c (.040) and TNF-α (< .001) were independent predictors of CTRP13 serum levels and HDL-C was independent positive (Table 3).

### Association of CTRP3 and CTRP13 serum levels with CAD

In case of CAD as dependent factor, logistic regression was performed in three condition, A: non-T2DM subjects, B: T2DM patients and C: all participants. Also, in each condition three models were conducted. First we performed univariate logistic regression for CTRP3 and CTRP13 serum levels as independent factors, the next models (A-1, B-1 and C-1) were adjusted for adiponectin, BMI and WHR and the final models (A-2, B-2 and C-2) were adjusted for all CAD risk factors (Table 4). CTRP3 showed a significant independent negative association with presence of CAD in whole study population, but in non-T2DM subjects, the association was lost after adjusting for CAD risk factors, and the association was not seen in patients with T2DM. CTRP13 serum level was independently and inversely associated with CAD in all above-mentioned models and in all three groups (Table 4).

**Table 4:** Odds ratios of CAD incident in Non-T2DM patients and T2DM patients according to CTRP3 and CTRP13 serum levels.

	CTRP3 serum levels			CTRP13 serum levels	
	OR (95% Cl)	P value	OR (95% Cl)	P value
**A-Non-T2DM Patients**				
Unadjusted	0.986(0.978–0.994)	**<0.001**	0.965 (0.951–0.980)	**<0.001**
Model A-1	0.989(0.980–0.998)	**0.023**	0.966 (0.948–0.984)	**<0.001**
Model A-2	0.994(0.981–1.008)	0.416	0.933 (0.889–0.979)	**0.005**
**B-T2DM Patients**				
Unadjusted	0.997 (0.991–1.004)	0.368	0.942 (0.918–0.967)	**<0.001**
Model B-1	0.998 (0.991–1.005)	0.527	0.941 (0.917–0.990)	**<0.001**
Model B-2	0.996(0.987–1.005)	0.425	0.940 (0.909–0.972)	**<0.001**
**C-All Subjects**				
Unadjusted	0.992(0.987–0.996)	**0.001**	0.959 (0.946–0.972)	**<0.001**
Model C-1	0.993(0.988–0.998)	**0.007**	0.959 (0.946–0.972)	**<0.001**
Model C-2	0.991(0.984–0.998)	**0.011**	0.940 (0.944–0.976)	**<0.001**

**Models A-1, B-1 and C-1**: adjusted for: Adiponectin, BMI and WHR

**Model A-2, B-2 and C-2**: adjusted for: sex, Age, BMI, WHR, Hypertension, LDL-C, HDL-C, TG, Smoking, IL-6, TNF-α and Adiponectin.

We also conducted logistic regression for 3-vessels disease as the dependent variable in CAD patients (group II and IV). In univariate models, there was no significant association between CTRP3 serum levels and 3-vessels disease. But CTRP13 serum levels showed a significant negative association with 3-vessels disease and this association remained after adjusting for adiponectin, BMI and WHR (Model 1) and also for CAD risk factors (model 2) (Table 5).

**Table 5:** Odds ratios of 3-vessels disease according to CTRP13 serum levels.

	CTRP13 serum levels	
	OR (95% Cl)	p
Unadjusted	0.976(0.961–0.992)	**0.003**
Model 1	0.978(0.962–0.994)	**0.007**
Model 2	0.971(0.951–0.992)	**0.006**

**Model 1**: adjusted for: Adiponectin, BMI and WHR

**Model 2**: adjusted for: Gender, Age, BMI, WHR, Hypertension, LDL-C, HDL-C, TG, Smoking, IL-6, TNF-α and Adiponectin

## Discussion

There is ample evidence showing plausible role of CTRPs in the pathogenesis of T2DM and CAD, since the expression of members of this family are dysregulated in metabolic diseases and obesity [24–28].

CTRP3 and CTRP13 are two new adipokines belonging to the CTRP family [3,6]. Specifically, CTRP3 is an anti-inflammatory adipokine which inhibits toll-like receptor (TLR) and nuclear factor κB (NF-κB) signaling pathway and also reduces IL-6 and TNF-α secretion [29,30]. Also, Peterson et al. have shown an immunomodulatory role for CTRP3 in systemic and chronic inflammation associated with insulin resistance and obesity, but no role in antagonizing LPS induced inflammation in mice model [31]. A more recent study reported a decrease in CTRP3 serum levels in stable and unstable angina pectoris patients [32]. In line with this, we showed that serum concentration of CTRP3 is lower in CAD patients with and without T2DM. We also illustrated for the first time that CTRP3 gene expression in PBMCs of CAD, T2DM and CAD+T2DM patients are decreased compared to the controls. PBMCs included mononuclear cells of immune system which are key contributors to atherosclerosis as the main underlying mechanism of CAD [33]. Several studies have shown that altered gene expression in PBMCs correlate with presence and extend of coronary artery disease [34]. It seems that PBMCs gene expression can be considered as a positive predictor of CTRP3 serum levels and therefore, it is tempting to speculate that PBMCs may be the potential sources of change in serum concentrations of CTRP3. Based on multivariate linear regression analysis, BMI and smoking appear to be negative predictors of the CTRP3 serum levels. Compatible with a previous study in obese patients [11,16] our finding is a further evidence linking obesity to CTRP3. It has been reported that cigarette decreases expression of PPAR-γ [35] and the CTRP3 promoter has a PPAR response element [20], hence it seems likely that negative association of smoking and CTRP3 may be a result of cigarette effect on PPAR-γ expression. Multivariate logistic regression shows the independent association between CTRP3 serum levels and the presence of CAD in non-T2DM subject and also in all participants. A previous study in patients without diabetes showed significant association of circulating CTRP3 with CAD [32]. In addition to non-T2DM, present study included T2DM patients. But in T2DM patients we found no association between CTRP3 and CAD, probably association with CAD was overshadowed due to effect of anti-diabetes medication especially metformin on CTRP3 serum levels [36]. Independent association between CTRP3 and CAD can be explained by the effect of CTRP3 on different aspects of atherosclerosis, such as inflammation and metabolic disorders. However, we found no correlation between CTRP3 and inflammatory markers, but association between CTRP3 and CAD may be justified by potent anti-inflammatory effect of CTRP3 on inhibiting inflammatory signaling pathways, reducing secretion of inflammatory mediators [29,37] and its favorable role in systemic and chronic inflammation associated with insulin resistance and obesity [31], as well as, cardio-protective effects of CTRP3 [10].

For the first time, we have reported decreased serum levels of CTRP13 in the CAD, T2DM and CAD+T2DM patients. CTRP13 has a significant negative correlation with elevated TG, TC and LDL-C and a positive correlation with HDL-C. Since reduced levels of HDL-C is an important aspect of dyslipidemia in T2DM as well as a classical and important risk factor for CAD [38], our findings in which HDL-C remain significant positive predictor of CTRP13 serum levels are of potential importance. However, further investigations are needed to clarify the relation between CTRP13 and above-mentioned parameters. It is generally accepted that TNF-α and IL-6 have important role in pathogenesis of T2DM and also atherosclerosis [33]. Importantly, CTRP13 serum levels have significant negative correlation with these two inflammatory adipocytokines, and in multivariate linear regression, TNF-α remains significant negative predictor of CTRP13 serum levels. In our study, we found BMI to be an independent negative predictor of CTRP13 serum levels. According to the previously mentioned findings, it is conceivable to speculate that serum levels of CTRP13 is probably down-regulated in the obesity and in inflammatory conditions. However, there is evidence that CTRP13 mRNA expressions are increased in obese mice [17]. This contradiction can be attributed to different nature of human and rodent studies. Based on multivariate models, we also showed that HOMA-IR and HbA1c among glucose metabolism parameters remain significant negative predictors of CTRP13 serum levels. Wei, Z et al demonstrated favorable effects of CTRP13 on glucose metabolism in insulin-mediated glucose uptake. It has been shown in detail that CTRP13 reduces gluconeogenesis in hepatocyte and also ameliorate insulin resistance via phosphorylation of AMPK in adipocytes, muscle cells and hepatocytes [17]. There is data on reducing adiponectin in obese human and possible involvement of increasing adiponectin in partial insulin sensitizer effect of thiazolidinedione [39]. Considering the effect of thiazolidinedione in CTRP13, upregulation of CTRP13, and correlation between CTRP13 and insulin resistance, it seems likely that CTRP13 can be considered as a part of complicated signaling pathway involving insulin sensitizer effect of thiazolidinedione. CTRP13 also had negative correlation with unfavorable lipids profile (TC, TG and LDL-C) and positive correlation with HDL-C. This suggests a possible link between CTRP13 and cholesterol and lipid metabolism. It seems likely that CTRP13 can affect lipid metabolism since CTRP13 induces phosphorylation of AMP-activated protein kinase (AMPK), thereby stimulating fatty acid oxidation [40]. Other studies also have illustrated that other CTRPs activate AMPK signaling pathway and augment fatty acid oxidation and inhibit acetyl CoA carboxylase (ACC) [41].

To our knowledge, we provide the first evidence of association of CTRP13 with the presence of CAD and also its significant association with 3-vessels disease as the severe stage of CAD disease. This correlation remained significant even after adjusting for the CAD risk factors. Association of CTRP13 with CAD and CAD severity corroborates previous studies regarding association of other CTRPs with CAD and atherosclerosis in humans [24,42] and further elucidate the role of CTRP13 as a possible contributory factor in pathogenesis of CAD and T2DM. However, future studies are needed to further clarify possible mechanisms.

Here, we showed CTRP13 serum levels have significant association with inflammatory factors, parameters of insulin resistance and cholesterol metabolism as the well-known CAD risk factors, suggesting CTRP13 may act as a part of complicated network involving the pathogenesis of CAD.

Our study has some limitations which must be considered. The first limitation is the relatively small sample size. Secondly, the design of our study is cross-sectional, which precludes drawing inferences about causality. Also, it should be noted that more large-scale clinical investigations using longitudinal data are necessary to confirm our data.

In conclusion, our study demonstrates an association between lower circulating levels of CTRP3 and CTRP13 with increased risk of CAD and T2DM, suggesting that these metabolic conditions are mediated at least in part through the effects of CTRP3 and CTRP13. However, more studies are required to dissect the roles of CTRPs in atherosclerosis and T2DM.

## Supporting Information

S1 Questionnaire English and Persian questionnaire. (DOCX)
