# Supplementary material for: Association of C1q/TNF-Related Protein-3 (CTRP3) and CTRP13 Serum Levels with Coronary Artery Disease in Subjects with and without Type 2 Diabetes Mellitus
Source: PLoS One. 2016 Dec 29;11(12):e0168773. doi: 10.1371/journal.pone.0168773 (PMC5199067; doi:10.1371/journal.pone.0168773)
Supplement: S1 Questionnaire — (DOCX) [file pone.0168773.s001.docx]

**English questionnaire**

**Patient details**

Patient code: ………………… File number: ……………… date: ………………

First name: …………… Last name: ……..…….. Sex: ⃝ Male ⃝ Female Date of birth: …/…/... (dd/mm/yyyy)

Weight: ……… (kg) height: …….. (cm) BMI: ……..

Waist circumference (cm): …… Hip circumference (cm): …… Waist to Hip ratio: ……

Contact number: …………………………

Home address:

**Smoking and Medical History**

In the past three months, have you smoked cigarette on a daily basis: ⃝ No ⃝ Yes

Please indicate below which disease(s) or condition(s) you have:

⃝ Myocardial Infraction ⃝ Stroke

⃝ Diabetes Mellitus *Type of diabetes:* ………………..

⃝ Kidney Disease *Specify type:* ………………………...

⃝ Arthritis or Rheumatic disease *Specify type:* ………………………..

⃝ Autoimmune disease *Specify type:* ………………………...

⃝ Liver Disease *Specify type:* ………………………...

⃝ Cancer *Specify type:* ………………………...

⃝ Infectious disease: *Specify type:* ………………………...

**Medications**

In the past six months, have you taken any medications: ⃝ No ⃝ Yes

If yes, please indicate which medications you took or you are taking currently:

………. ………. ………. ………. ………. ………. ………. ………. ……….

………. ………. ………. ………. ………. ………. ………. ………. ……….

**Persian questionnaire**

**مشخصات بیمار**

کد بیمار: .......... شماره پرونده: .............. تاریخ: ............

نام: ................ نام خانوادگی: ............... جنس: ⃝ آقا ⃝ خانم تاریخ تولد (روز/ماه/سال): .../..../...

وزن (کیلوگرم): .......... قد (سانتی متر): ........... شاخص توده بدنی: ............

دور کمر(سانتی متر): ........ دور لگن (سانتی متر): .......... نسبت دور کمر به دور لگن: ........

تلفن: .............................

آدرس منزل:

**سابقه مصرف سیگار و سوابق پزشکی**

آیا در سه ماه گذشته روزانه سیگار مصرف می‌کرده‌اید: ⃝ خیر ⃝ بله

لطفاً مشخص کنید کدامیک از بیماری‌ها یا شرایط زیر را داشته‌اید:

⃝ سکته قلبی ⃝ سکته مغزی

⃝ دیابت شیرین نوع دیابت: ........................

⃝ بیماری کلیوی: نوع بیماری: ......................

⃝ آرتریت یا بیماری روماتوئیدی نوع بیماری: ......................

⃝ بیماری خودایمن نوع بیماری: ......................

⃝ بیماری کبدی نوع بیماری: ......................

⃝ سرطان نوع بیماری: ......................

⃝ بیماری عفونی نوع بیماری: .....................

**مصرف دارو**

آیا در شش ماه گذشته داروی مصرف کرده‌اید: ⃝ خیر ⃝ بله

اگر مصرف کرده‌اید، لطفا نام داروها را بگوئید:

............ ............ ............ ............ ............ ............ ............ ........... ...........

............ ............ ............ ............ ............ ............ ............ ........... ...........
